# Errata

**Published:** 2009-04

In Table 1 of the article by Tarantini et al. [ Environ Health Perspect 117:217–222 (2009)], the sequence for the *iNOS* forward primer should be AATGAGAGTTGTTGGGAAGTGTTT instead of AATGAGAGTTGTTGTTGGGAAGTGTTT.

The authors apologize for the error.

In the article “Diesel Exhaust Particles Activate the Matrix-Metalloproteinase -1 Gene in Human Bronchial Epithelia in a β-Arrestin–Dependent Manner via Activation of RAS” by Li et al. [ Environ Health Perspect 117:400–409 (2009)], the competing financial interest declaration was incorrect. Jinju Li was not supported by the Philip Morris grant but by a Leon-Goldberg Fellowship. Therefore, the declaration should be as follows:

S.A.S and W.L. received funding from Philip Morris USA and Philip Morris International. The other authors declare they have no competing financial interests.

In the February 2009 Focus article [“Carbon Offsets: Growing Pains in a Growing Market,” Environ Health Perspect 117:A62–A68 (2009)], a quotation at the bottom of p. A64 is incorrectly attributed to David Antinioli. The quotation should actually be attributed to Bill Burtis of Clean Air-Cool Planet; Antinioli is affiliated with the Voluntary Carbon Standard Association. On p. A68 of the same article, a quotation by James Lovelock is incorrectly attributed to the 22 January 2009 issue of *New Scientist*; the quotation actually appeared in the 24 January 2009 issue.

The December 2008 Focus article [ “The Yuck Factor: When Disgust Meets Discovery,” Environ Health Perspect 116:A524–A527 (2008)] incorrectly stated on p. A525 that Fountain Valley, California, is north of Redwood City. Fountain Valley is actually south of Redwood City.

*EHP* regrets the errors.

